# Grammatical feature dissimilarities make relative clauses easier: A comprehension study with Italian children

**DOI:** 10.1016/j.lingua.2010.03.018

**Published:** 2010-09

**Authors:** Flavia Adani, Heather K.J. van der Lely, Matteo Forgiarini, Maria Teresa Guasti

**Affiliations:** a Department of Psychology, University of Milano-Bicocca, Italy; b Department of Psychology, Harvard University, USA; c Department of Linguistics, University of Potsdam, Germany

**Keywords:** Relative clause comprehension, Italian acquisition, Number, Gender

## Abstract

The Relativized Minimality approach to A′-dependencies (Friedmann et al., 2009) predicts that headed object relative clauses (RCs) and which—questions are the most difficult, due to the presence of a lexical restriction on both the subject and the object DP which creates intervention. We investigated comprehension of center-embedded headed object RCs with Italian children, where Number and Gender feature values on subject and object DPs are manipulated. We found that, Number conditions are always more accurate than Gender ones, showing that intervention is sensitive to DP-internal structure. We propose a finer definition of the lexical restriction where external and syntactically active features (such as Number) reduce intervention whereas internal and (possibly) lexicalized features (such as Gender) do so to a lesser extent. Our results are also compatible with a memory interference approach in which the human parser is sensitive to highly specific properties of the linguistic input, such as the cue-based model (Van Dyke, 2007).
